# From fear to facts: a multi-channel approach to information seeking amid influenza-like illness outbreaks

**DOI:** 10.3389/fpubh.2025.1545942

**Published:** 2025-03-24

**Authors:** Shenghao Qi, Jen Sern Tham, Moniza Waheed, Norliana Hashim

**Affiliations:** ^1^Department of Communication, Faculty of Modern Languages and Communication, Universiti Putra Malaysia, Selangor, Malaysia; ^2^Brain and Mental Health Research Advancement and Innovation Networks (PUTRA BRAIN), Universiti Putra Malaysia, Selangor, Malaysia

**Keywords:** influenza-like illness (ILI), risk information seeking and processing model (RISP), multichannel information seeking, information-seeking intentions, China

## Abstract

**Background:**

During recurrent large-scale influenza-like illness (ILI) crises, the factors influencing the information-seeking intentions of Chinese individuals across multiple channels during crises remain underexplored.

**Objective:**

Guided by the risk information seeking and processing (RISP) model, this study proposes a modified RISP model to comprehensively analyze information-seeking intentions through the lens of risk communication.

**Methods:**

To empirically validate the proposed research model, we conducted an online cross-sectional survey with 2,604 Chinese citizens aged 18 years and older. Structural equation modeling (SEM) and ordinary least squares regression analysis were employed to analyze the survey data.

**Results:**

Our findings revealed that during ILI crises, Chinese individuals experienced a spectrum of emotions; as perceived risk increased, negative emotions intensified while positive emotions decreased. Increased negative emotions correlated with a greater sense of information insufficiency, whereas heightened positive emotions correlated with a reduced perception of it. Consequently, Chinese individuals facing information deficiencies were more inclined to seek information from diverse sources, including interpersonal sources, traditional media, search engines, and social media. Moreover, statistical analysis indicated that stronger beliefs in channel complementary strengthened the relationship between information insufficiency and information-seeking intention across multiple channels (access to medical expertise belief, tailorability belief, convenience belief, anonymity belief).

**Conclusion:**

This study outlines a pathway for advancing the RISP model and offers practical strategies for effective risk communication to mitigate risks and enhance public perception and behavior. It also discusses implications for health communication, promotion, and behavior change.

## 1 Introduction

China’s rapid advancements in digital technologies have transformed its information landscape, allowing for personalized media consumption and diverse information channels (1). This evolution is especially relevant in the context of recent influenza-like illness (ILI) outbreaks, where effective information dissemination is critical for public health response (2). Influenza is a highly contagious and deadly virus, and its complications can cause significant morbidity and mortality (3). Globally, seasonal influenza results in approximately one billion cases annually, contributing to 290,000–650,000 respiratory deaths annually (4). Like other countries worldwide, ILI poses significant public health challenges in China, particularly during the flu season (2, 5). The government faces a heavy burden from influenza each year, with approximately 88,000 excess deaths attributed to the disease (5).

In recent years, several ILI epidemics have occurred in China (2). Influenza has risen to its highest level during the same period in China in the last decade, and it continues to increase (6). China continues encountering formidable challenges in preventing and controlling influenza (2). To address these challenges, the Chinese government has leveraged various traditional and social media communication platforms, such as WeChat, Weibo, and Douyin^1^, to disseminate up-to-date information and advisories on ILI to the public (7). Therefore, authentic influenza-related information has become central to the Chinese government’s establishment of a public governance mechanism for emergencies within the public health domain.

The emergence of digital media and the subsequent evolution of traditional media have altered people’s news consumption patterns, leading to channel plurality, where individuals access news across multiple platforms (1). The emergence of digital media and the subsequent evolution of traditional media have altered people’s news consumption patterns, leading to channel plurality, where individuals access news across multiple platforms (8, 9). Previous research suggests that a lack of information in critical situations prompts individuals to seek more in-depth information from diverse sources (10). However, existing studies on risk communication primarily focus on general information-seeking behavior and have not explored how the dynamic media environment influences public information-seeking during crises (11–14). Only a few studies have investigated the roles of different media channels in risk information acquisition (9, 15). Therefore, it is crucial to examine how Chinese residents utilize multiple channels to seek risk information during an ILI pandemic.

**Figure 1:**
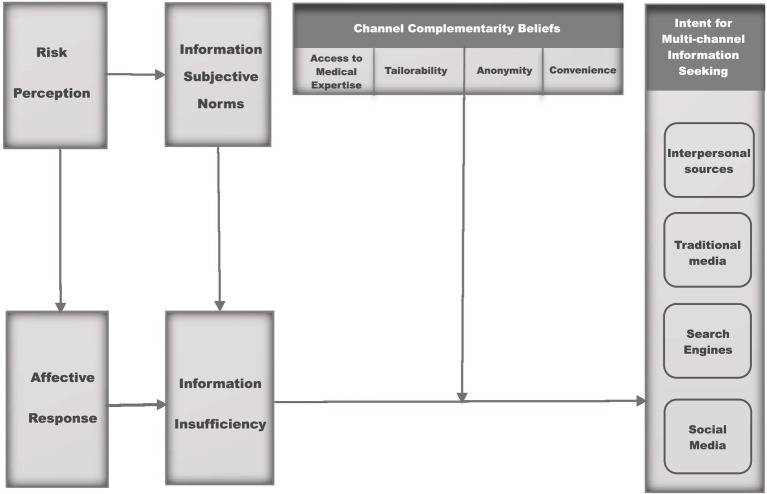
Hypothesized model.

Considering the aforementioned, this study aims to identify the patterns of channel usage among Chinese residents when seeking information related to the risk of influenza-like illness (ILI) and to assess the theoretical applicability of the modified Risk Information Seeking and Processing (RISP) model within the context of ILI awareness and behavior among this population (Figure 1). The following sections will delve into the key concepts underpinning this research and provide evidence supporting the proposed pathways.

## 2 Theoretical framework

The RISP model is an overarching theoretical framework explaining how individuals seek and process information related to perceived risks or hazards. It illustrates the principal determinants predisposing individuals to systematically and thoughtfully seek and process pertinent risk information (16). It delineates cognitive and social psychological variables that elucidate individuals’ information-seeking and processing behaviors toward specific environmental or health hazards (17–19). Previous research has applied numerous theories and models in investigating the role of information communication during critical situations, such as the Health Belief Model (HBM) (20) and the Extended Parallel Process Model (EPPM) (21). However, the RISP model integrates theories from multiple disciplines, such as information science and cognitive psychology (16). This enables it to understand individuals’ information behaviors and their risk responses from a broader perspective (16). Additionally, it can better adapt to different types of risk information and individual differences (16, 22). Therefore, this study contends that the RISP model is more suitable for dealing with crises such as Influenza-like illnesses. According to Griffin and colleagues (10, 17, 18, 23) (1999–2013), individuals tend to seek information when they believe that their current knowledge is insufficient, a perception shaped by both their understanding of the risks involved and their emotional responses. Information subjective norms (ISN) play a role in raising awareness of these information gaps, especially during crises, while the characteristics of the perceived risk often evoke strong emotional reactions. Personal attributes also come into play, influencing both ISN and risk perceptions. Additionally, the effect of information insufficiency on the desire to seek more information is moderated by relevant channel belief (RCB), which helps determine how individuals respond to perceived risks.

In recent years, the application of the RISP model has broadened to address various risks, including health-related concerns, environmental crises, disasters, and product safety issues. Researchers like Yang, Jin, and Zhou, among others, have explored its relevance across these different areas, significantly providing key insights into health intervention programs (12–14). Moreover, successful validations of the model have been conducted in both Eastern and Western cultural contexts, confirming its versatility and broad applicability (9, 13, 24, 25). Through these extant studies, the RISP model provides a valuable framework for understanding how people manage risk information in a wide range of scenarios (16, 22). For example, numerous studies have effectively employed the RISP model to examine individuals’ information seeking behavior in the context of the COVID-19 pandemic (coronavirus disease 2019) (11, 12), as well as during the H1N1 outbreak (26). The RISP model principles can also be applied to Influenza-Like Illnesses (ILIs), providing valuable insights into how individuals navigate information during such health crises (24, 27, 28). By applying this model, researchers can better understand public health communication strategies that can effectively address the challenges posed by ILI outbreaks and ultimately improve health outcomes (24, 29). Therefore, it is essential to examine how residents in China seek multichannel risk information employing the RISP model during an ILI pandemic (Figure 1). This exploration will contribute to developing targeted communication strategies that resonate with the public’s information-seeking behavior and enhance overall public health response during epidemics.

More than 100 empirical studies have employed the RISP model, demonstrating robust support for most of its hypothesized relationships (22). However, this model has faced criticism, highlighting areas that require further refinement and development (22). First, scholarly discourse suggests that future research should employ more robust probability sampling techniques and incorporate large national samples for more accurate model estimation (16). Furthermore, using a reliable and internally consistent set of multiple indicators to assess key variables could enable researchers to evaluate the entire model through structural equation modeling (16).

Second, research has predominantly focused on the relationship between public risk perception and the elicitation of intense negative emotions, such as worry, fear, and anger, particularly in the context of crises and pandemics (11, 12). This focus has often overshadowed the role of positive emotions, which also emerge during critical situations (30). Scholars highlights the need for further investigation into how positive emotions impact information-seeking in critical situations, emphasizing the importance of exploring this dynamic in future research (30).

Third, empirical support for the significance of relevant channel belief (RCB) in the RISP model remains tenuous, and the specific beliefs constituting RCB remain unclear (19). Critics argue that the RCB construct is underdeveloped, with issues in its conceptualization and operationalization, including inconsistencies and incomplete definitions (16). While RCB is positioned as a moderating factor in the original RISP model (18), empirical studies on its moderating role are scarce (9, 24). Several RISP-based studies have excluded RCB (12, 14, 15) or treated it as an exogenous variable (13, 25). These ambiguities, along with measurement challenges, impede the coherent development of RISP-based research (16, 19). Given this gap, it is crucial to reconceptualize RCBs within the RISP model, particularly in the context of multichannel information seeking. This study integrated a particularly relevant construct derived from the channel complementarity theory- channel complementarity beliefs - to strengthen the focus of this moderator, making the concept clearer and complete (31).

Fourth, scholars have critiqued the measurement of information insufficiency and sufficiency thresholds in RISP models, as these constructs are often assessed with single-item measures, which may undermine predictive power (15). Furthermore, while the original RISP model incorporates multichannel information seeking (10), empirical studies on this aspect remain limited. Scholars have called for greater attention to information-seeking behaviors within the model (16). Recent trends indicate a shift from examining specific channels to considering information needs more holistically (32). Future research could further explore how individuals seek information across multiple sources (12). Consequently, adapting the RISP model to better account for contemporary multichannel information-seeking behaviors is highly beneficial. The conceptualization of each pathway is examined in the following section.

## 3 Study hypotheses

### 3.1 Linking risk perception to affective responses

Risk perception encompasses individuals’ evaluations of the characteristics and severity of risks (23). Various studies on the RISP model have explored different dimensions of risk perception (16). Nevertheless, a composite variable that combines both perceived severity and perceived susceptibility (i.e., perceived severity × perceived susceptibility) has emerged as the most consistently incorporated dimension across these studies (10, 16, 23). Perceived severity refers to the extent to which an individual believes that the harm associated with the risk is serious, while perceived susceptibility denotes the degree to which an individual perceives themselves as likely to experience harm from the risk (17). Empirical findings have found that risk perception significantly influences affective responses (17, 23). Affective response refers to an individual’s general feelings toward a stimulus, which can be positive or negative (10, 16, 18). Previous researchers have explored negative affective responses to risk exposure, such as fear, anger, sadness, and anxiety building (12, 16). These emotions are common during crises and pandemics, including the ILI epidemic (11, 28). For instance, the COVID-19 pandemic has significant catastrophic potential, leading to heightened risk perceptions and strong affective responses, such as fear, anger, and anxiety (33). Studies utilizing the RISP model indicate that both negative (e.g., anger, fear) and positive (e.g., hope, optimism) responses influence individuals’ sense of information insufficiency and their information-seeking and processing behaviors (11, 23).

Risk perception triggers strong negative emotions such as worry, fear, and anger, with a significant correlation identified (17, 23, 34). Increased risk perception correlates with heightened negative affective reactions, particularly evident in contexts like the COVID-19 pandemic (24, 27). Researchers suggest that both negative and positive affective responses to risk, including anger, worry, and optimism, influence information seeking and processing (23, 35). Furthermore, positive emotions like hope has explanatory power in the RISP model (32). While Lu et al. (28) found a negative relationship between risk perception and positive impacts in geothermal system crises, Zhou et al. (14) reported a negative correlation among risk severity, sensitivity, judgment, and positive affective responses, supporting this hypothesis. Given the previously mentioned validation of positive and negative emotions evoked by people during health epidemics such as COVID-19 (11, 12), this study proposed the following hypotheses:

*H1a:* Risk perception positively affects negative affective responses.

*H1b:* Risk perception negatively affects positive affective responses.

### 3.2 Linking risk perception to information subjective norms

According to the concept of subjective norms in the TPB (36), ISN describes individuals’ social expectations for specific risks. In other words, people believe that significant others (i.e., family members) expect them to know or be informed about the degree of a particular risk (18). Within the context of ILI research, subjective norms denote the social pressures individuals perceive from significant others (e.g., family, friends, colleagues) concerning their awareness and understanding of the associated risks. Risk perception may influence how strongly individuals adhere to subjective norms during the context of an ILI epidemic. For example, suppose an individual perceives a high risk of ILI and realizes their peers and community leaders are advocating for vaccination. In that case, they may feel more social pressure to get vaccinated (37). Moreover, people often align their behavior with the norms and expectations of their social groups. If heightened risk perception aligns with strong social norms promoting preventive behaviors (e.g., vaccination and hand hygiene), individuals are more likely to follow these practices (38). A significant positive correlation was observed between risk perception and informational subjective norms (25), exemplified by Chinese parents who, perceiving higher risks regarding vaccine safety, feel a greater expectation of staying informed about vaccine-related issues. Based on this observation, this study formulates the following hypothesis:

*H2:* Risk perception positively affects ISN.

### 3.3 Linking affective response to information insufficiency

Information insufficiency is a core component of the RISP model, grounded in the sufficiency principle of the HSM, which posits that individuals strive to achieve sufficient confidence in their information processing (39). Information insufficiency depicts an individual’s need for information, essentially epistemic motivation (15). Consequently, individuals are driven to thoroughly process information until they feel confident in the validity of their judgments (40). Previous tests of the RISP model revealed that negative affective responses, such as worry and anxiety, contribute to information insufficiency; greater negative emotions correlate with increased feelings of insufficiency (15, 17, 23). Conceptually, negative affective responses may promote information insufficiency by motivating cognitive responses to the target, while positive affective responses may increase information insufficiency by increasing personal relevance, expanding personal attention to new information, or both (15, 18, 30). For example, the researchers pointed out that when individuals experience disease-related anxiety and fear, they become aware of the inadequacy of their current disease-related knowledge (24). They believe that they need more information and will make the necessary efforts to reach a sufficient level of confidence to effectively manage risks (24). In a recent empirical study, negative affective responses toward COVID-19 trigger people’s need for information sufficiency, and stronger negative emotions lead to heightened perceptions of insufficiency (27). This has also been confirmed in hurricane disasters, where those who experience intense negative emotions seek to find more information about the impacts of hurricanes (13). Enlighteners should consider public affective responses and mitigate negative emotions stemming from information overload (24, 27). Furthermore, although most researchers have not explored the relationship between positive affective responses and information insufficiency (13, 24, 27), some scholars have still made attempts. For example, Ford et al. (11) explored the negative correlation between positive emotions and information insufficiency during the COVID-19 crisis. When individuals experience strong positive emotions regarding the epidemic, they are more likely to feel that they possess sufficient information, thus reducing their perception of information inadequacy (41). In keeping with Yang and Kahlor (32) exploration of the impacts of both positive and negative affect on information insufficiency. Taking into account the two categories of affective responses triggered by the Influenza-Like Illness (ILI) epidemic in the current Risk Information Seeking and Processing (RISP) framework, this study formulated the following hypotheses:

*H3a:* Negative affective responses positively affect information insufficiency.

*H3b:* Positive affective responses negatively affect information insufficiency.

### 3.4 Linking information subjective norms to information insufficiency

Greater social pressure to be aware of risks increases individuals’ likelihood of seeking information. Individuals’ ISN influences their perception of information insufficiency (10). While ISN directly affects information seeking, it also exerts an indirect influence through information insufficiency. Higher subjective norms for information may lead to increased information seeking (18, 23). Individuals who perceive social desirability in understanding a risk topic often view their knowledge as insufficient, prompting them to seek additional information (27). Recent empirical studies have reported that ISN is positively related to information insufficiency (27, 34). Subjective norms for information about the COVID-19 pandemic are negatively related to information sufficiency (24). During the Spring Festival of 2020, when the COVID-19 pandemic broke out, discussions with relatives and friends strengthened subjective norms for information (27). Consequently, this study derives the following hypothesis:

*H4:* ISN positively affects information insufficiency.

### 3.5 Linking information insufficiency to intent for multichannel information seeking

The RISP model incorporates the dimension of multichannel information seeking and proposes that information insufficiency can stimulate information seeking to represent varying degrees of breadth and depth (10). Research indicates that perceived information insufficiency significantly influences active information seeking (9, 23, 40), functioning as a core variable that motivates individuals to seek risk information. Factor analysis identifies two dimensions of information seeking: information avoidance and information seeking (32). Empirical evidence, particularly in health contexts, supports the positive correlation between information insufficiency and risk information seeking, as exemplified during influenza-like outbreaks when public knowledge is limited (42). Several studies have examined multichannel information seeking as a research variable (15). However, most focus on general information-seeking behavior without investigating how individuals utilize various media channels. In the contemporary all-media environment, it is essential to consider media usage patterns. Researchers like Moreno et al. (43) and Zhang et al. (44) underscore the importance of exploring diverse media for information collection, particularly during health epidemics. Therefore, this study proposed the following hypothesis:

*H5:* Information insufficiency positively affects multichannel risk information-seeking intentions.

### 3.6 The moderating effect of channel complementary beliefs on the relationship between information insufficiency and multichannel risk information seeking intention

Initially, relevant channel belief (RCB) was defined as personal beliefs that a particular information channel contains relevant, unbiased, and trustworthy information (10). In today’s information environment, source trust is increasingly crucial (13). RCB refers to “the mix of cognitive and affective ways in which people evaluate information channels” (45). The beliefs constituting the RCB are unclear (19). Researchers have noted that RCBs are the least RISP predictors in RISP models, possibly because of the difficulty in finding consistent conceptualizations and operationalizations (16, 18). The few studies that have examined this variable have also produced inconsistent results (18). The original RISP model hypothesized that RCBs moderate the effects of information insufficiency on information seeking and processing (10, 15). In addition, Jin and Lane (12) examined the risk communication during COVID-19 in the United States; RCB moderated the effect of information insufficiency on information-seeking intentions. Specifically, positive beliefs about communication channels led to increased information seeking when faced with insufficiency. However, most researchers have not identified a significant relationship between RCB and information-seeking intentions (19, 23). Ruppel and Rains (31) proposed that the information-seeking process can systematically incorporate information sources based on four complementary characteristics, namely, access to medical expertise, tailorability, anonymity, and convenience. In this context, CCT and RCBs discuss the characteristics of information sources (17, 18, 31). Compared with the media characteristics of RCB, channels with more accurate descriptions of channel complementary characteristics are more suitable for the current media ecological environment and the audience’s media information seeking. Given the communication mode and characteristics of ILI information, RCB was reconstructed, and this study proposes the following research hypotheses:

*H6a:* Access to medical expertise belief of multiple channels moderates the relationship between information insufficiency and multichannel information-seeking intention.

*H6b:* Tailorability belief of multiple channels moderates the relationship between information insufficiency and multichannel information-seeking intention.

*H6c:* Convenience belief of multiple channels moderates the relationship between information insufficiency and multichannel information-seeking intention.

*H6d:* Anonymity belief of multiple channels moderates the relationship between information insufficiency and multichannel information-seeking intention.

## 4 Methods

### 4.1 Recruitment

**Figure 2:**
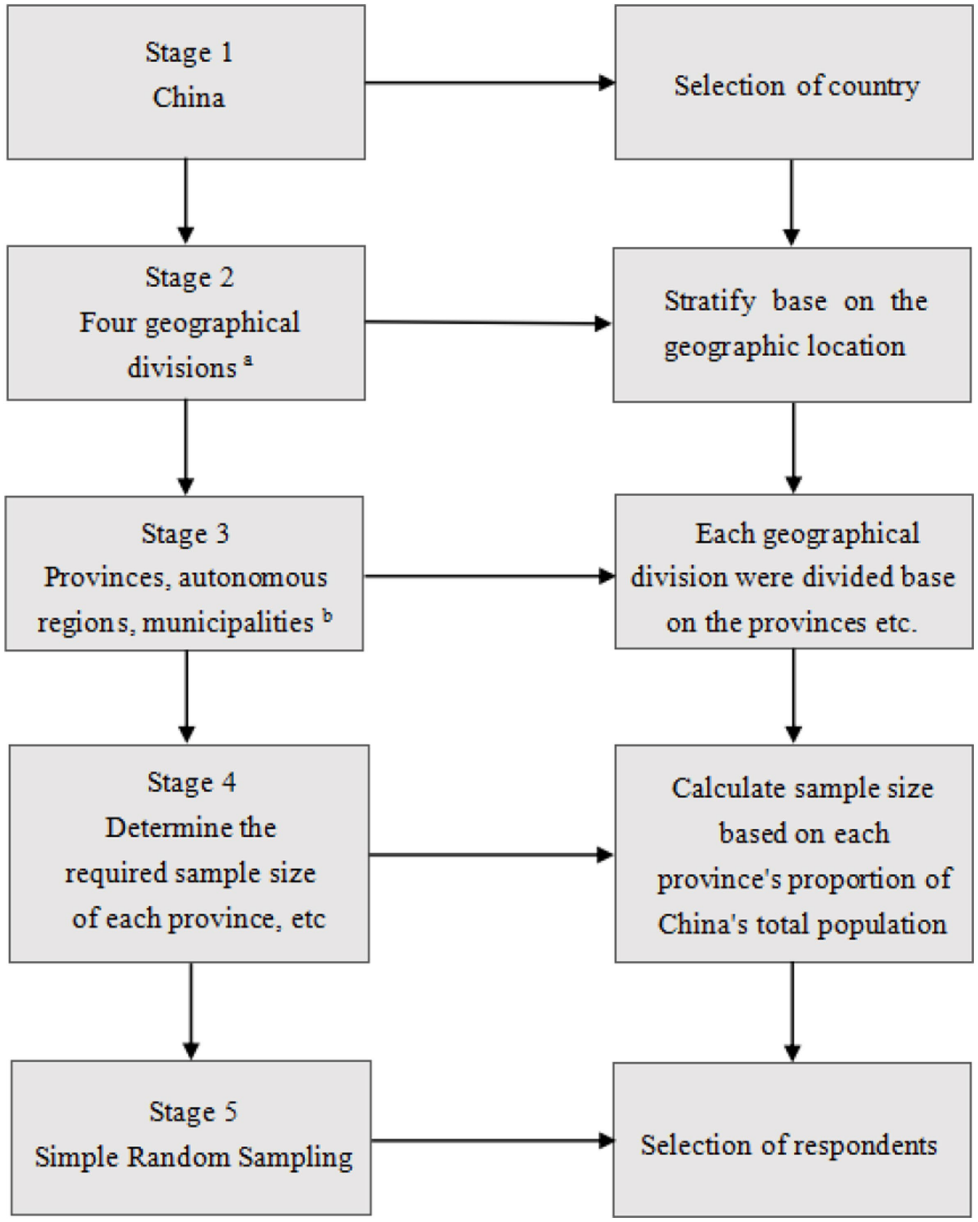
The multi-stage sampling procedure. ^a,b^ China National Bureau of Statistics (47).

**Figure 3:**
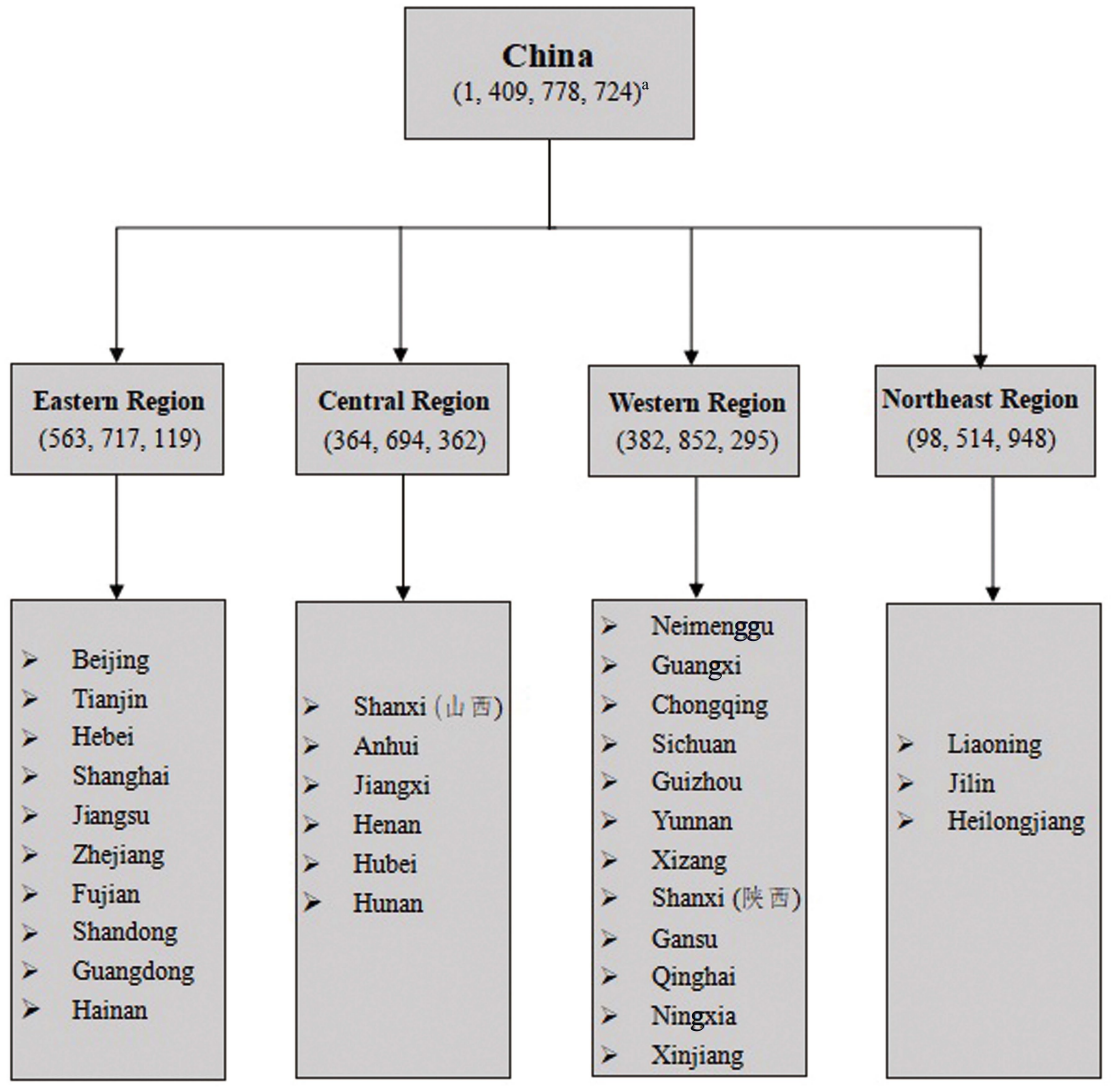
Stratified sampling in China. ^a^ Data were obtained from China’s Seventh National Census (52). The Chinese population refers to the population of the 31 provinces, autonomous regions, and municipalities on the mainland, excluding active military, Hong Kong, Macao, and Taiwan residents and foreigners living in the 31 provinces, autonomous regions, and municipalities.

This study used a cross-sectional survey design with a nationwide online approach to capture a comprehensive snapshot of the Chinese population. A multi-stage sampling method with five stages was employed (Figure 2) (46). In Stage 1, the researcher defined the Chinese population as including the 31 provinces, autonomous regions, and municipalities of Mainland China, excluding active military personnel and the Special Administrative Regions of Hong Kong, Macao, and Taiwan. Stage 2 utilized the regional classification from the China National Bureau of Statistics (47), dividing China into eastern, central, western, and northeastern regions. In Stage 3, these regions were further categorized into specific provinces and municipalities based on Bureau data (2021) (47) (Figure 3). Stage 4 involved calculating the proportion of each administrative division within China’s total population, which was essential for determining the minimum sample size required from each area to ensure demographic representation. Finally, Stage 5 entailed the random selection of samples from each division according to the predetermined sample sizes, ensuring the sample’s representativeness and statistical validity.

The sample size was determined using the A-priori Sample Size Calculator for SEM (48), which recommended a minimum of 1,888 participants. The required sample size for each of the 31 localities was calculated based on their proportion of the Chinese population. The study targeted Chinese residents aged 18 and older, recognized as fully capable of exercising civil rights and responsibilities (49). Data were collected through a reputable third-party survey company known for its expertise in Chinese social science research (50, 51). We independently developed and translated the questionnaire from English to Chinese. Data collection occurred between May 26, 2023, and June 12, 2023.

This study analyzed valid responses from 2,604 participants (*N* = 2,604) aged 18–70 years (mean = 31.26, median = 31.00, SD = 7.39), with 50.8% males and 49.2% females, reflecting gender proportions from the China Statistical Yearbook (52). Over half of the participants held university degrees (73%), with 13.1% from colleges and 7.6% with graduate or higher education. The respondents were from all 31 Chinese provinces, autonomous regions, and municipalities, with the sample size in each region meeting the required proportions. Salary ranges varied (mean = 9,012.70 RMB, median = 8,000.00 RMB, SD = 10,466.73 RMB), with the majority (89.7%) earning between 1,000 and 15,000 RMB.

### 4.2 Ethical considerations

This research was approved by the ethical review board (Ethic Committee for Research Involving Human Subjects, JKEUPM) of Universiti Putra Malaysia in 2023. The reference number for this approval is JKEUPM-2023-1197.

## 5 Measures

We developed bilingual (English and Chinese) survey questionnaires based on previous literature, with modifications to align with the research context. The survey included seven sections, with six variables and Demographic Profiles (a control/confounding variable) as the final section. Four experts pre-tested the questionnaire for validity, and a pilot test with 30 Chinese respondents showed Cronbach’s *α* values ranging from 0.74 to 0.90, confirming the instrument’s reliability.

### 5.1 Risk perception

Risk perception measurement includes four items: individuals and their families, their local community, the whole of China, and everyone in the world (40). Each item was measured on a 6-point scale (1 = not at all, 6 = a great deal) and referenced previous studies (32, 40). Respondents were asked to indicate how much the influenza-like illness harmed or threatened them; four items assessed perceived severity, and four items measured perceived susceptibility to influenza-like illness. A composite risk perception score was calculated by multiplying severity by susceptibility (23, 40). Higher scores indicated stronger risk perception (mean = 4.89, median = 5.00, SD = 0.85).

### 5.2 Affective response

The modified RISP model included two types of affective responses: negative (fear, anger, sadness, anxiety) (12, 25, 28, 53) and positive (hope, optimism, elevation) (12, 25, 28). Respondents rated their feelings toward influenza-like illness on a 6-point scale (1 = strongly disagree, 6 = strongly agree). Composite scores for positive and negative affective responses were calculated by summing and averaging the ratings. Higher scores indicated more intense negative affective responses (mean = 4.29, median = 4.5, SD = 0.92) or positive affective responses (mean = 1.95, median = 1.67, SD = 0.99).

### 5.3 Information subjective norms (ISN)

The measurement of ISN encompasses five items: me, my friends, most people, my family, and others (13, 40, 54). Respondents rated their expectations of learning about influenza-like illness from the different subjects on a 6-point scale (1 = strongly disagree, 6 = strongly agree). The composite score was obtained by summing and averaging the ratings, with higher scores indicating greater ISN (mean = 4.8, median = 5.00, SD = 0.72).

### 5.4 Information insufficiency

The measurement of information insufficiency, adapted from previous scales (13), asked respondents to rate their need for information about influenza-like illness on a 6-point scale (1 = strongly disagree, 6 = strongly agree). Two items assessed information insufficiency: (1) “I need to know everything about the risks of influenza-like illnesses” and (2) “I need more information about the risks of influenza-like illnesses.” A composite score was calculated by summing and averaging the two items, with higher scores indicating greater information insufficiency (mean = 5.10, median = 5.0, SD = 0.71).

### 5.5 Channel complementarity beliefs

Channel complementarity beliefs were measured in four categories: access to medical expertise, tailorability, anonymity, and convenience, with two questions per category based on previous research (55). Items were rated on a 6-point scale (1 = strongly disagree, 6 = strongly agree). The scores for each belief were summed and averaged to create a composite variable, with higher scores indicating stronger channel complementarity beliefs (mean = 4.5, median = 4.53, SD = 0.52).

### 5.6 Intent for multi-channel information seeking

This research categorized multi-channel information-seeking intentions into four types: interpersonal, traditional media, search engine, and social media (9, 15, 29). Respondents rated their attention to influenza-like illness information from these channels. Interpersonal sources included family, friends, co-workers, and doctors; traditional media encompassed newspapers, TV, magazines, and radio; search engines included sites like CCTV, People’s Daily, and major portals like Wangyi; social media included platforms like WeChat, microblogs, TikTok, and mHealth apps. Information-seeking intention was measured on a 0 to 10 scale (0 = none, 10 = a lot), and a composite score was calculated. Higher scores indicated a stronger intention to seek information from these channels (mean = 7.13, median = 7.25, SD = 1.34).

### 5.7 Demographics

The survey questionnaire collected demographic data on respondents’ gender, age, salary, education qualification (56), occupation (57), and province, which were examined as control variables. Except for age and salary, all variables were nominal measurements. The instrument is provided in Appendix 1.

### 5.8 Data analysis

The data analysis began with Structural Equation Modeling (SEM) to assess model fit. Prior to SEM, confirmatory factor analysis (CFA) was conducted on the measurement model to evaluate construct validity. Following CFA, SEM was performed using the maximum likelihood method to test the revised RISP model’s standardized coefficients. Model fit was evaluated using multiple indices: relative chi-square (CMIN/DF), comparative fit index (CFI), Tucker–Lewis index (TLI), normed fit index (NFI), incremental fit index (IFI), root mean square error of approximation (RMSEA), and standardized root mean square residuals (SRMR). Acceptable fit criteria include CMIN/DF < 5, CFI, TLI, NFI, and IFI > 0.9, and RMSEA and SRMR <0.08 (58, 59). The SEM model hypothesis was tested by examining the standardized coefficients of the pathways between observed variables (58). The final step involved conducting an ordinary least squares regression to assess the moderating effect of the channel, as outlined in the conceptual framework. Moderation was demonstrated by testing whether the interaction terms between the moderating and independent variables significantly predicted the dependent variables (60).

## 6 Results

### 6.1 Structural equation model (SEM) and path coefficients

**Figure 4:**
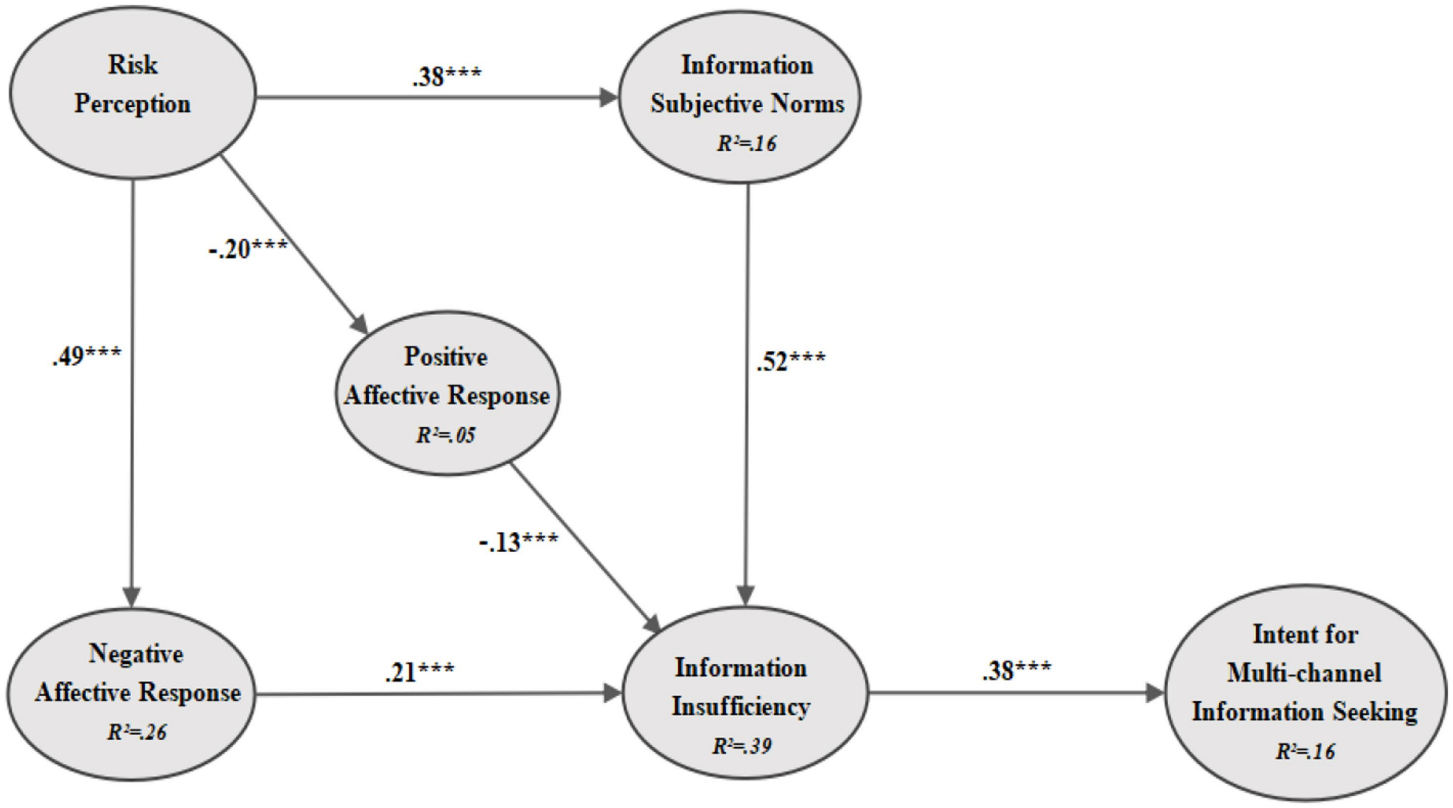
Simplified diagram of the SEM of RISP (*N* = 2,604). Significance key: ***p < 0.001 (*N* = 2,604) and *R*^2^ = squared multiple correlations.

Controlling for demographics variables, the structural model showed an acceptable fit (CMIN/DF = 4.48; CFI = 0.96; TLI = 0.94; NFI = 0.94; IFI = 0.96; RMSEA = 0.037; SRMR = 0.04). As illustrated in Figure 4, the results indicated that risk perception had a positive impact on negative affective responses (*β* = 0.49, *p* < 0.001); risk perception had a negative effect on positive affective responses (*β* = −0.20, *p* < 0.001), thus supporting Hypothesis 1a and Hypothesis 1b. Additionally, risk perception positively affected ISN (*β* = 0.38, *p* < 0.001), confirming Hypothesis 2. Moreover, the study found that negative affective responses positively affected information insufficiency (*β* = 0.21, *p* < 0.001), and positive affective responses had a negative effect on information insufficiency (*β* = −0.13, *p* < 0.001), supporting Hypothesis 3a and Hypothesis 3b. Furthermore, the test results reported that ISN positively affected information insufficiency (*β* = 0.52, *p* < 0.001), thereby supporting Hypothesis 4. Finally, the SEM test results demonstrated that information insufficiency positively affected intent for multichannel information seeking (*β* = 0.38, *p* < 0.001), supporting Hypothesis 5.

### 6.2 Ordinary least square (OLS) regression and moderating testing

To assess the moderating effect, this study employed OLS regression using STATA software (version 17) (Table 1). The results indicate that access to medical expertise beliefs significantly moderated the relationship between information insufficiency and intent for multi-channel information seeking.

**Table 1:** Channel complementarity beliefs of multichannel.

	(1)	(2)	(3)	(4)
	Intent for Multi-channel Information Seeking	Intent for Multi-channel Information Seeking	Intent for Multi-channel Information Seeking	Intent for Multi-channel Information Seeking
Information insufficiency	0.34^***^^a^	0.38^***^	0.43^***^	0.54^***^
	(9.45)^b^	(10.82)	(12.27)	(14.98)
c_xm1	0.22^***^			
	(3.85)			
m1^c^	0.93^***^			
	(19.52)			
c_xm2		0.23^***^		
		(4.30)		
m2^d^		0.74^***^		
		(18.50)		
c_xm3			0.25^***^	
			(5.49)	
m3^e^			0.59^***^	
			(17.43)	
c_xm4				0.17^***^
				(3.67)
m4^f^				0.23^***^
				(6.97)
_cons	0.95^***^	1.74^***^	2.33^***^	3.40^***^
	(3.96)	(8.04)	(11.63)	(15.81)
*N*	2,604	2,604	2,604	2,604
adj. *R*^2^	0.21	0.20	0.20	0.11

a *p < 0.05, **p < 0.01, and ***p < 0.001.

b T statistics in parentheses.

c m1 = access to medical expertise belief of multichannel.

d m2 = tailorability belief of multichannel.

e m3 = convenience belief of multichannel.

f m4 = anonymity belief of multichannel.

Individuals with higher levels of information insufficiency (*β* = 0.34, *t* = 9.45, *p* < 0.001) and stronger beliefs in medical expertise (*β* = 0.93, *t* = 19.52, *p* < 0.001) exhibited greater intent to seek multi-channel information. The interaction term (*β* = 0.22, *t* = 3.85, *p* < 0.001) confirms that access to medical expertise beliefs amplifies the effect of information insufficiency on multi-channel information-seeking intention.

**Figure 5:**
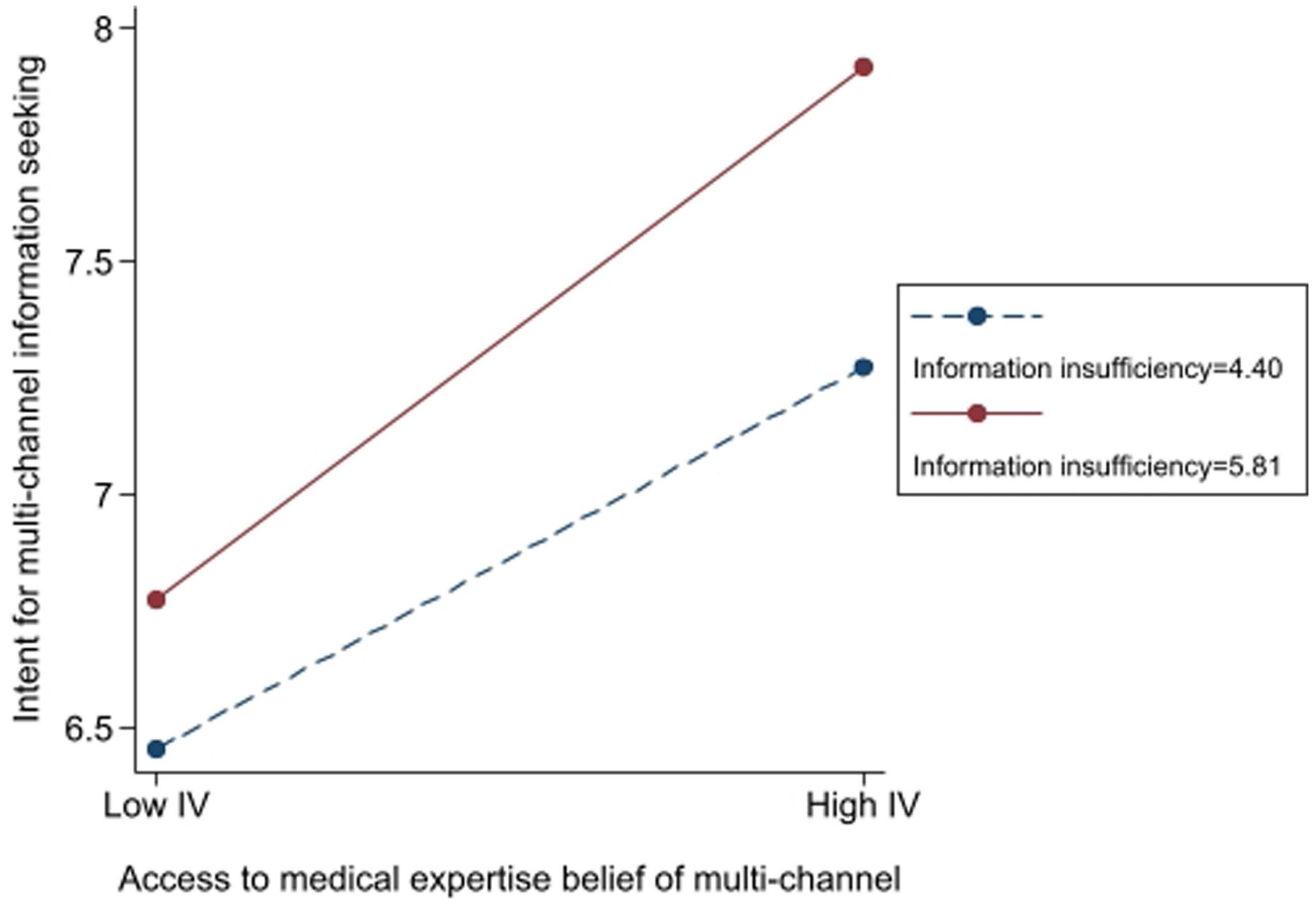
The moderating role of access to medical expertise beliefs in the effect of information insufficiency on the intent for multichannel information seeking.

To illustrate the practical significance, a simple slope analysis (Figure 5) reveals that when belief in medical expertise is high (+1 SD), the effect of information insufficiency on seeking intention increases by 64.7% compared to when belief in medical expertise is low (−1 SD). This suggests that access to medical expertise not only facilitates greater information-seeking behavior but also significantly strengthens the reliance on multi-channel sources when individuals perceive information insufficiency.

Figure 5 presents a graphical representation of the moderation effect, showing the standardized coefficients for different levels of access to medical expertise beliefs. Additionally, Table 1 includes key evaluation indicators such as *R*^2^ changes, confidence intervals, and effect sizes to provide a more comprehensive view of model performance.

From a theoretical perspective, these findings align with the Risk Information Seeking and Processing Model, which posits that perceived information insufficiency drives information-seeking behaviors, and this effect is contingent upon moderating factors such as trust in expertise. The results underscore the importance of expert credibility in shaping individuals’ risk information-seeking strategies across multichannel. Thus Hypothesis 6a was supported.

Additionally, the findings showed that beliefs regarding tailorability significantly moderated the relationship between information insufficiency and intent for multi-channel information seeking. Individuals with higher levels of information insufficiency (*β* = 0.38, *t* = 10.82, *p* < 0.001) and higher tailorability belief (*β* = 0.74, *t* = 18.50, *p* < 0.001) demonstrated a stronger intention to seek information across multiple channels. The interaction term (*β* = 0.23, *t* = 4.30, *p* < 0.001) indicates that tailorability beliefs enhance the effect of information insufficiency on multi-channel information-seeking intention.

**Figure 6:**
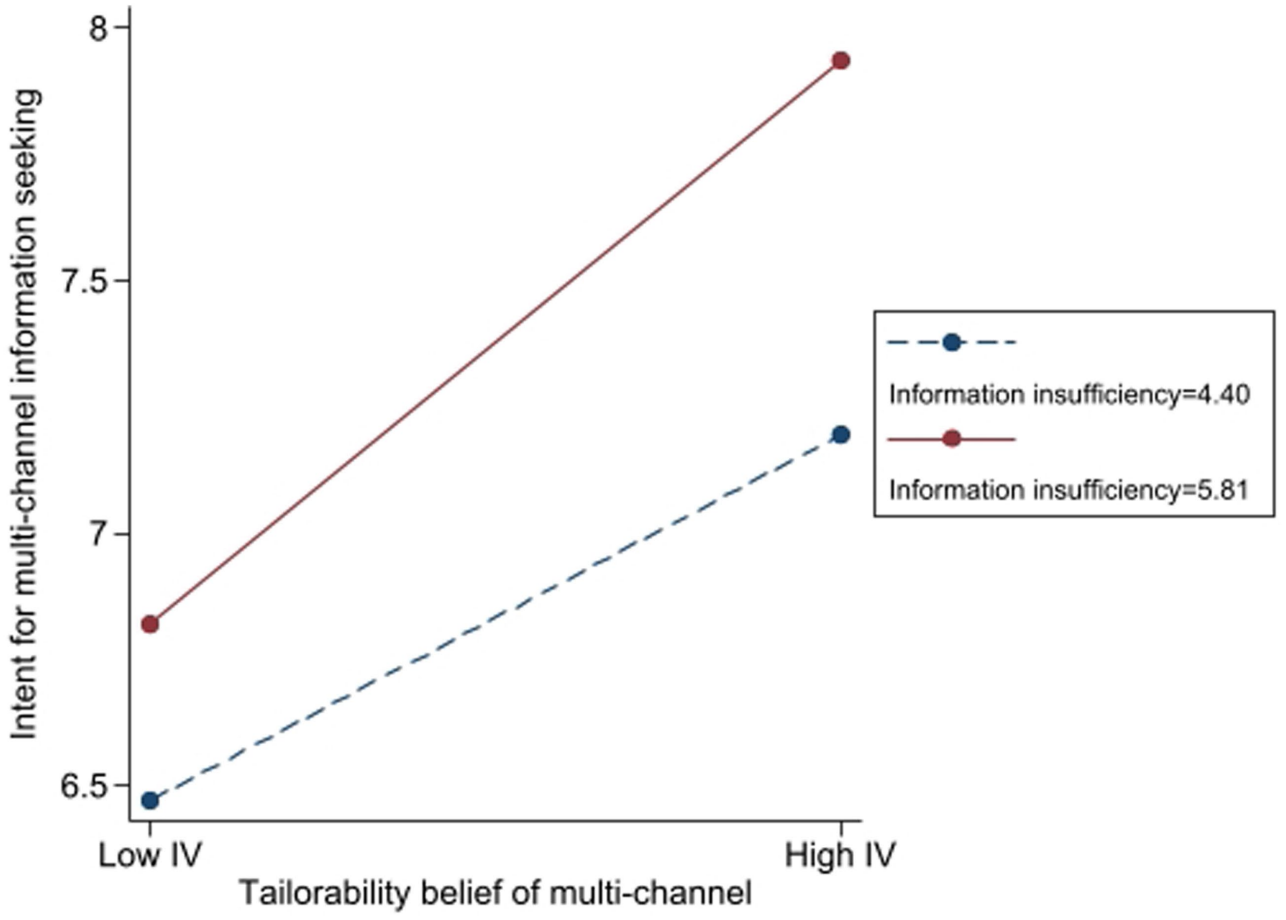
The moderating role of tailorability belief in the effect of information insufficiency on intent for multichannel information seeking.

To demonstrate the practical significance, a simple slope analysis (Figure 6) shows that when belief in tailorability is high (+1 SD), the effect of information insufficiency on information-seeking intention increases by 67.6% compared to when belief in tailorability is low (−1 SD). This finding suggests that tailorability not only encourages greater information-seeking behavior but also significantly amplifies the reliance on multi-channel sources when individuals perceive information insufficiency.

Figure 6 illustrates the moderation effect, displaying the standardized coefficients across varying levels of tailorability beliefs. Furthermore, Table 1 presents key evaluation metrics, including changes in *R*^2^, confidence intervals, and effect sizes, offering a more thorough assessment of the model’s performance. From a theoretical standpoint, these findings are consistent with the Risk Information Seeking and Processing Model, which asserts that perceived information insufficiency motivates information-seeking behaviors, with this effect being moderated by factors such as tailor information. The results highlight the critical role of tailorability in influencing individuals’ risk information-seeking strategies across multiple channels, confirming support for Hypothesis 6b.

Furthermore, the results revealed that convenience-related beliefs significantly moderated the relationship between information insufficiency and intent for multi-channel information seeking. Participants with higher levels of information insufficiency (*β* = 0.43, *t* = 12.27, *p* < 0.001) and stronger beliefs in convenience (*β* = 0.59, *t* = 17.43, *p* < 0.001) presented greater intent to seek information through multiple channels. The interaction term (*β* = 0.25, *t* = 5.49, *p* < 0.001) confirms that convenience beliefs strengthen the effect of information insufficiency on multi-channel information-seeking intention.

**Figure 7:**
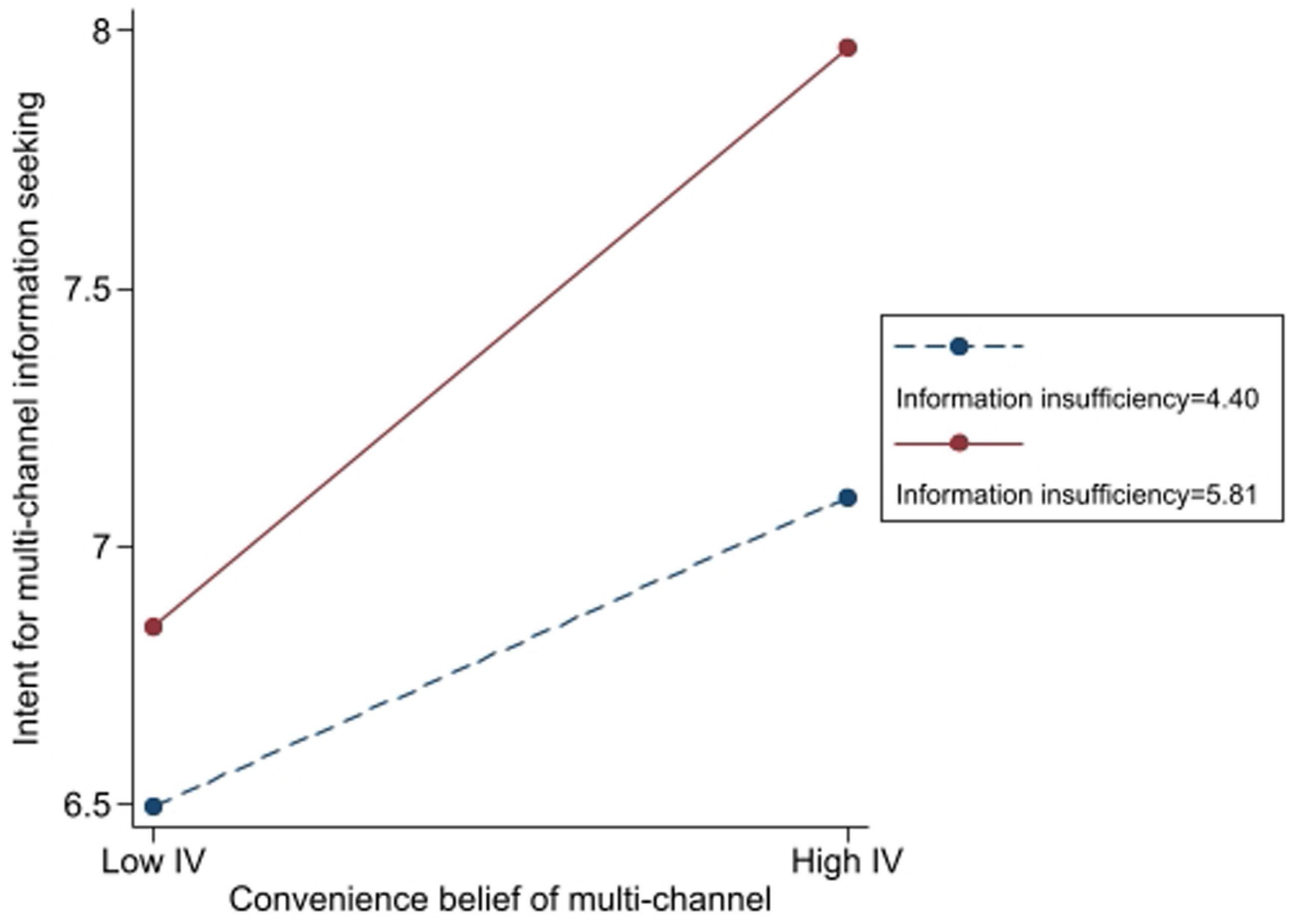
The moderating role of convenience belief in the effect of information insufficiency on intent for multichannel information seeking.

To elucidate the practical implications, a simple slope analysis, as depicted in Figure 7, demonstrates that when an individual’s belief in convenience is high (+1 SD), the effect of information insufficiency on the intention to seek information escalates by 73.5% relative to the scenario where belief in convenience is low (−1 SD). This finding implies that convenience not only promotes more extensive information-seeking behavior but also substantially enhances the dependence on multiple information channels when individuals perceive a lack of information.

Figure 7 graphically illustrates the moderation effect, depicting the standardized coefficients for varying degrees of beliefs regarding convenience. Moreover, Table 1 encompasses crucial evaluation metrics, including *R*^2^ changes, confidence intervals, and effect sizes to provide a more comprehensive view of model performance.

From a theoretical perspective, these findings align with the Risk Information Seeking and Processing Model, which posits that perceived information insufficiency drives information-seeking behaviors, with this effect being contingent upon moderating factors such as ease of access and utilization. The results emphasize the importance of convenience in shaping individuals’ risk information-seeking strategies across multiple channels, thereby supporting Hypothesis 6c.

Lastly, the analysis demonstrated that beliefs concerning anonymity also significantly moderated the relationship between information insufficiency and intent for multi-channel information seeking. Participants with higher levels of information insufficiency (*β* = 0.54, *t* = 14.98, *p* < 0.001) and stronger anonymity belief (*β* = 0.23, *t* = 6.97, *p* < 0.001) indicated greater intent to seek multi-channel information. The interaction term (*β* = 0.17, *t* = 3.67, *p* < 0.001) confirms that anonymity beliefs enhance the effect of information insufficiency on the intention to seek information across multiple channels.

**Figure 8:**
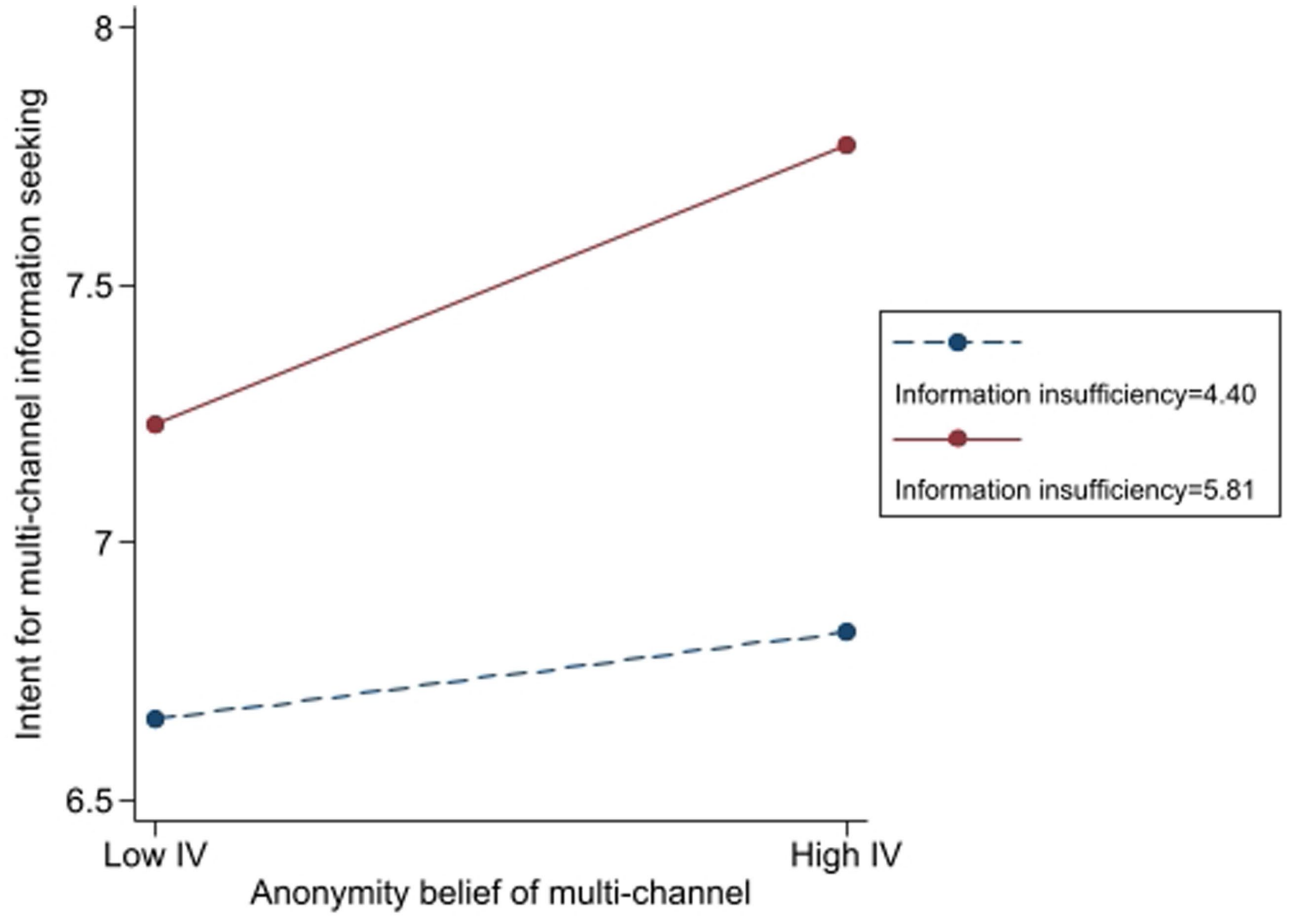
The moderating role of anonymity belief in the effect of information insufficiency on the intent for multichannel information seeking.

To demonstrate the practical significance, a simple slope analysis (Figure 8) shows that when belief in anonymity is high (+1 SD), the effect of information insufficiency on information-seeking intention increases by 50% compared to when belief in anonymity is low (−1 SD). This suggests that anonymity not only encourages greater information-seeking behavior but also significantly amplifies the reliance on multi-channel sources when individuals perceive information insufficiency.

Figure 8 provides a graphical representation of the moderation effect, displaying the standardized coefficients across varying levels of anonymity beliefs. Additionally, Table 1 presents key evaluation indicators, including changes in *R*^2^, confidence intervals, and effect sizes, offering a more comprehensive assessment of model performance.

**Figure 9:**
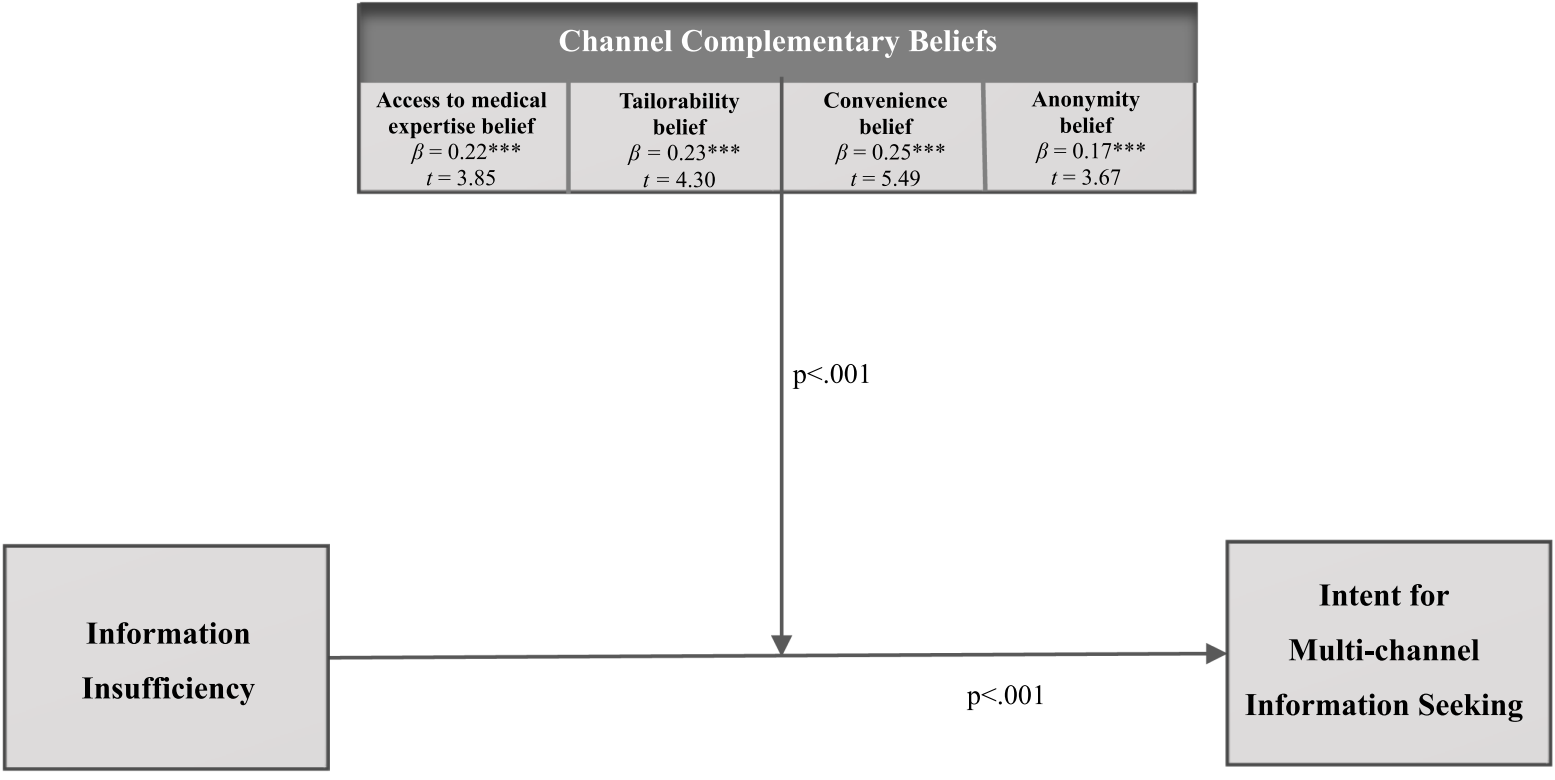
Simplified diagram of the OLS of intent for multichannel information-seeking group (*N* = 2,604). Significance key: ***p < 0.001 (*N* = 2,604).

From a theoretical perspective, these findings align with the Risk Information Seeking and Processing Model, which posits that perceived information insufficiency drives information-seeking behaviors, with this effect being contingent upon moderating factors such as the ability to obtain information without revealing one’s identity. The results highlight the importance of anonymity in shaping individuals’ risk information-seeking strategies across multiple channels, thus supporting Hypothesis 6d. The results of the OLS tests for the intent of the multichannel information-seeking group are presented in Figure 9.

## 7 Discussion

### 7.1 Principal findings

During crises, individuals typically turn to a variety of information sources to manage uncertainty and make informed decisions. This study examines the factors influencing Chinese individuals’ intentions to seek information across multiple channels, drawing on the RISP model. The findings demonstrate that risk perception, affective responses, information subjective norms, channel complementarity beliefs, and information insufficiency shape significant roles in shaping individuals’ information-seeking behaviors, particularly within the unique sociocultural and media landscape of China. Specifically, this study identifies multichannel information-seeking related to ILI risks among Chinese residents, with a focus on assessing the applicability of the modified RISP model to such behaviors. The results indicated that Chinese residents actively seek ILI-related information during outbreaks through various channels, including interpersonal communication, traditional media, search engines, and social media. It highlights a complementary relationship among these channels, reflecting their interrelated use in information-seeking behavior regarding ILI. This aligns with Rains and Ruppel (55), who found that individuals utilize multiple media channels to gather information on specific topics and support Zhang et al. (44), which demonstrated that the availability of information from diverse sources significantly influences information-seeking behaviors.

Risk perception and affective responses play critical roles in determining how individuals engage with crisis-related information. These results demonstrated that risk perception has a significant positive effect on negative affective responses and a significant negative effect on positive affective responses. These results align with prior research. Kim et al. (53) noted that heightened risk perception during the COVID-19 pandemic triggered negative emotions, such as sadness and anxiety. In contrast, low-risk perception during a crisis encourages optimism and sympathy (61). Specifically, in China, where there is a widespread emphasis on family and individual health, a high perceived risk of influenza-like illnesses leads to concerns for personal and family well-being, resulting in negative emotions like fear and anxiety (62). Pandemics disrupt daily life through measures such as school closures and transportation restrictions, heightening negative emotions due to uncertainty (61, 63). Media coverage and community control measures during crises can amplify these perceptions, further triggering negative emotional responses (64). Higher perceived risk increases the likelihood of negative emotional reactions. To curb the pandemic, social distancing and reduced activities can lead to loneliness and diminished positive emotions (65). Increased risk perception may also prompt cautious consumer behavior, causing operational and economic challenges for businesses, heightening anxiety and reducing positive emotions (66). Additionally, uncertainty about the pandemic’s duration can further undermine positive emotions (66). During an ILI outbreak, higher perceived risk among individuals in China is linked to a reduced likelihood of positive emotional responses.

This study also reveals that risk perception significantly influences information subjective norms (ISN), consistent with previous studies (25). Yang and Liu (25) found that Chinese parents’ perceptions of vaccine safety risks notably shaped social expectations to seek information during a crisis. In China, heightened sensitivity to health risks amplifies risk perception, especially after major public health events (67). Concerns over ILI, transmission risks, and their societal impact increase individuals’ vigilance, thereby increasing their risk perception (62). Additionally, the Chinese government disseminates accurate epidemic information and control measures through various channels during ILI epidemics, promoting public understanding of risks and guiding the public to correctly understand the risks (7). The media’s intense focus on epidemics further pressures individuals to actively seek information to manage risks (7). When risk perception is high, people are more likely to urgently seek information on disease characteristics, transmission, and prevention, reinforcing their subjective norms regarding information-seeking (68).

Moreover, the results show that negative affective responses significantly increase information insufficiency, while positive affective responses decrease it. These findings align with previous research (19, 23). For instance, Park et al. (24) found that negative affective responses during the COVID-19 outbreak significantly influenced perceptions of information insufficiency. Conversely, positive affective responses were linked to stronger perceptions of information insufficiency, as noted by researchers in the context of COVID-19 risk communication (11). In the case of an ILI crisis in China, heightened negative emotions, such as worry over personal and family health or anxiety about the future, often lead to increased attention and demand for information (28, 63). This heightened concern can create a perception of insufficient information, such as an unclear understanding of transmission, severity, and prevention (28, 63). In contrast, when negative emotions are less intense, individuals may perceive the available information as sufficient (62, 68). Additionally, timely and accurate information from government and health authorities during ILI outbreaks can reduce information insufficiency by instilling confidence and generating positive emotions (62). Consequently, intense positive emotions reduce the perception of information inadequacy (41).

Social influence is another critical factor shaping information-seeking behaviors. The findings highlight information subjective norms significantly influences perceptions of information insufficiency, supporting findings from prior risk communication research (13, 53). For instance, Park et al. (24) and Li and Zheng (27) demonstrated that adherence to social norms during the COVID-19 pandemic heightened perceptions of information insufficiency. During an ILI epidemic in China, a strong societal focus on public health generates public pressure regarding the acquisition of information (69). Additionally, the uncertainty surrounding the epidemic, such as virus mutations and symptom variability, exacerbates feelings of information insufficiency despite efforts to gather information (70, 71). Information overload, stemming from diverse and often conflicting sources, compounds this perception (71). Thus, the pressure to acquire information amplifies the sense of inadequacy regarding available information (70, 71).

The most direct predictor of multi-channel information-seeking intention is information insufficiency, which remains a central construct in the RISP model. This study found that information insufficiency significantly enhances the intention to seek information through multiple channels. This aspect can be analyzed at two levels. First, information insufficiency directly affects information needs, aligning with findings from risk communication research (16). For instance, Lee et al. (42) demonstrated that a lack of knowledge about viruses drives people to seek information. Second, this study’s results support the RISP model’s emphasis on multichannel information seeking (10) and confirm its relevance in today’s communication landscape (9, 31). In the context of China, during an ILI outbreak, people often experience worry and uncertainty (68). When information is insufficient, individuals first turn to family, friends, and colleagues due to the cultural value placed on interpersonal relationships and trust in familiar sources (72). They seek information on symptoms, prevention, and treatment from close contacts. In Eastern cultures, trust is often built on emotional bonds and a sense of control over intimate social connections. In contrast, in Western cultures, trust tends to be rooted in broader ethical principles and formal networks (73, 74). A comparative study of Chinese and German cultures, representing interdependent and independent cultural orientations respectively, found that Chinese individuals displayed higher levels of interpersonal trust and shared more objective information within close relationships than their German counterparts (72). Next, Chinese individuals rely on traditional media for authoritative information, as these channels offer timely updates, control measures, and expert advice during epidemics (1, 75). Additionally, search engines have become vital sources of information in China, especially during an ILI outbreak. When information is lacking, individuals use search engines to find relevant data, including symptoms, treatments, and prevention methods (76, 77). Search engines provide vast amounts of information, which users can filter and organize according to their needs (1, 8). Lastly, with its large user base, social media plays a critical role in information-seeking behavior (76, 78). Users search for relevant topics and follow groups related to ILI, leveraging social media’s (such as Weibo and WeChat) rapid dissemination and interactivity to exchange experiences, opinions, and information (8, 76).

Another key finding relates to the moderating effect, as this research demonstrates that channel complementarity beliefs (access to medical expertise, tailorability, anonymity, and convenience) significantly moderate the relationship between information insufficiency and multichannel information-seeking intentions (interpersonal sources, traditional media sources, search engine sources, and social media channels). This finding aligns with previous research suggesting that relevant channel beliefs moderate the link between information insufficiency and information-seeking intentions (18). While few recent studies have examined this variable, few have validated its moderating role (9, 12). For instance, during COVID-19, Jin and Lane (16) found that when individuals perceive information channels as credible and valuable, their intention to seek additional information increases with perceived insufficiency. Conversely, other studies have tested the main effect of channel beliefs without exploring their moderating impact or found no support for the hypothesized moderating relationship (19, 24). Notably, the field of risk communication has yet to incorporate modifications to channel beliefs related to communication channels. This study successfully demonstrates the moderating effect of channel complementarity beliefs.

This study presents innovative findings on channel beliefs, showing that the refined processing of these beliefs aligns with audience cognition and expectations in risk contexts. It also confirms the applicability of channel complementarity to relevant channel beliefs in ILI risk communication. The results support previous work suggesting a need to reformulate the composition of relevant channel beliefs (16). In China, trust in medical experts is generally high (79). During epidemics, individuals who believe they can access medical expertise through certain channels are more likely to seek information (80). For example, authoritative health TV programs featuring medical experts can enhance viewers’ trust in the advice they receive (81). Similarly, online medical platforms offering expert consultations may increase individuals’ willingness to use these channels if they perceive expert insights are available (8). Additionally, Chinese society values practicality and operability. Individuals who believe they can obtain actionable strategies for managing influenza-like illnesses through various channels are more likely to seek multi-channel information (82). Public health accounts offering specific preventive measures and dietary advice can encourage individuals to use these channels if the information is perceived as applicable (8). Government-issued guidelines motivate individuals to use multiple channels to understand and apply protective strategies (7). In China’s online environment, privacy concerns may deter some from using specific channels (83). However, during an epidemic, channels that guarantee anonymity can encourage use, as individuals feel more comfortable obtaining information without revealing their identity (84). This belief in anonymity can alleviate psychological stress and encourage individuals to actively use these channels to address information deficiencies (33). Finally, the rapid development of Internet and mobile technologies in China has increased the demand for convenient information access (1). During epidemics, individuals are more likely to rely on channels perceived as convenient, such as smartphone apps and social media platforms (such as Weibo and WeChat), which provide timely updates and expert advice (76, 85).

Taken together, these findings highlight the complex interplay between psychological, social, and contextual factors in shaping crisis-related information-seeking behaviors. The results suggest that effective crisis communication strategies in China should account for how risk perception, emotional responses, and social influences drive individuals toward multi-channel information-seeking. Ensuring that credible information is readily available across diverse platforms can help manage public uncertainty and reduce reliance on misinformation. Moreover, leveraging trusted social figures and peer networks can enhance the dissemination of reliable information while addressing the collective nature of information-seeking in Chinese society. By understanding these dynamics, policymakers, media practitioners, and crisis communication experts can develop more effective strategies to guide public information-seeking behaviors in times of crisis.

## 8 Implications for research and practice

The findings of this study uncover a rich narrative that significantly contributes to the field of risk communication in public health crises, serving as a foundational reference, advancing theoretical understanding, and offering empirical validation in this critical area.

First, from a theoretical viewpoint, this study broadens the scope of the extended RISP model’s application by exploring its relevance to a more nuanced domain: widespread respiratory illnesses (i.e., ILI). Furthermore, the significance of incorporating a more comprehensive set of variables has been confirmed, thereby enhancing the robustness of the modified RISP model. In contrast to previous studies that focused on examining a narrow set of variables derived from the original model, this study undertakes refinement by integrating a broader set of dynamic variables. This expansion provides a more nuanced understanding of the model’s application and effectiveness. For example, the application of the RISP model encompasses the analysis of multichannel information, which differs from earlier studies that predominantly examined a single channel. This study categorizes multichannel information-seeking intentions into distinct domains: interpersonal, traditional media, search engine, and social media. This nuanced division aligns with the contemporary media landscape and reflects the evolving patterns of public information-seeking behavior. Existing studies have often overlooked the complexity of public emotional responses during crises, which are not exclusively negative. In contrast, this study introduces a more nuanced approach by categorizing affective responses into positive and negative. This classification enables a more precise analysis of emotional reactions and their effects on the extended RISP model.

Most studies have extracted a few variables to adjust and validate models in new research contexts (16, 22). Few researchers have engaged in innovative theoretical integration of the RISP model. Herein, verification of the channel complementarity belief variable demonstrates that replacing RCB with a more comprehensive, specific, and detailed moderator can improve the effectiveness of multichannel information strategies. The revised moderating variable, RCB, has adeptly integrated concepts from channel complementarity theory, thereby enriching its conceptual framework. The moderating effect of channel complementarity beliefs is investigated through two distinct analytical lenses: general channels and subchannels. Subchannels include interpersonal information channels, traditional media channels, search engine channels, and social media channels. The findings offer substantial and innovative insights into risk communication, as delineated within the RISP model.

Second, the findings provide insights into practical implications for public health crisis response and risk management. In situations of frequent ILI occurrences, the provision of correct and effective guidance on disease prevention behaviors and response measures through the communication of crisis information is the mission of current risk communication. The results elucidate the interrelationships among variables in the modified RISP model and provide a framework for organizing and implementing strategies and interventions. According to this study, the Chinese public utilized multiple channels to seek information during an ILI crisis. This behavior indicates that practitioners should promote the communication of high-quality and credible information through an expanded range of channels. In addition, risk communicators and government officials must address the public’s emotional states because negative and positive emotional responses from the public during an ILI crisis can influence perceptions of information insufficiency. Efforts should be made to foster positive affective responses to enhance accurate comprehension of the crisis and mitigate negative affective responses. Moreover, the public’s perception of various information channels—access to medical expertise, tailorability, anonymity, and convenience—positively influences their information-seeking intention during an ILI crisis. Therefore, risk communication practitioners should prioritize shaping these beliefs when presenting information channels to enhance individuals’ willingness to engage with and utilize these channels. Positive, active, and powerful information from various channels can alleviate anxiety and uncertainty, reduce the perception of information insufficiency, and improve individuals’ ability to manage risks. Consequently, a positive and active communication cycle can be achieved. The research results can strengthen people’s literacy in fighting risks, improve modern health crisis management, cater to residents’ medical and health needs, and keep up with China’s health strategy, ultimately contributing to the “Healthy China 2030 Plan” and providing feasible directions.

### 8.1 Limitations and suggestions for future research

The limitations of this study are evident in its conceptual and methodological dimensions. First, this study only measured people’s intention to seek information from multiple channels. However, it did not predict the actual information-seeking behavior of the respondents, including the relationship between information insufficiency and information avoidance or systematic processing. In the original RISP model, these variables served as measures and responses to the effects of communication. From this perspective, incorporating these variables into measurement provides data support for further applied research. Second, this study is based on cross-sectional survey data for hypothesis testing; therefore, it cannot make rigorous claims about causal relationships between variables. This study recommends that future studies use experimental data (dividing subjects into a control group and an experimental group for investigation) or a longitudinal design (conducting two waves of surveys on the same timeline with the same respondents) to test the causal relationship in the RISP model. Third, this study is a risk communication survey of residents in China, which represents the Eastern cultural worldview, although previous studies have generally supported the applicability of the RISP model to different cultural backgrounds (16, 22, 53). However, the applicability of the modified RISP model to Western countries remains to be verified in future research in these regions. The author looks forward to the general applicability of the modified RISP model across various cultural backgrounds.

## 9 Conclusion

This study measured the severity of the crisis caused by frequent ILI and recognized that understanding the information-seeking behavior of Chinese residents is a valuable step toward meeting the public’s information needs during a crisis, mitigating its impact on residents, and providing guidance for combating crises. A new crisis communication plan, a revision of the RISP model, and a predictive study on the information-seeking intentions of Chinese residents must be developed. The driving factors include risk perception, affective responses, ISN, information insufficiency, and channel complementary beliefs. The modified RISP model achieved a good fit standard after testing, which is broadly consistent with previous RISP models. The modified model describes a vivid process map of multichannel information-seeking intentions. The research results are presented to researchers and practitioners, offering reference indicators for promoting work at the audience and communication levels, demonstrating the explanatory value of the RISP model, and proposing feasible directions for model adjustment.

## Data Availability

The raw data supporting the conclusions of this article will be made available by the authors, without undue reservation.

## Footnotes

^1^Douyin is the Chinese version of TikTok’s short video application, and both belong to China’s ByteDance company.

## Glossary

ILI: influenza-like illness
RISP: Risk Information Seeking and Processing
ISN: information subjective norm
RCBs: relevant channel beliefs
RCB: relevant channel belief
China CDC: Chinese Center for Disease Control and Prevention
WHO: World Health Organization
CDC: Center for Disease Control and Prevention
COVID-19: coronavirus disease 2019
CNNIC: China Internet Network Information Center
CSM: CSM Media Research
HSM: heuristic–systematic model
CCT: channel complementarity theory
TPB: theory of planned behavior
SEM: structural equation modeling
CFA: confirmatory factor analysis
CMIN/DF: relative chi-square
CMIN: Chi-square
DF: degree of freedom
CFI: comparative fit index
TLI: Tucker–Lewis index
NFI: normed fit index
IFI: incremental fit index
RMSEA: root mean square error of approximation
SRMR: standardized root mean square residuals
OLS: ordinary least squares
