# Supplementary material for: From fear to facts: a multi-channel approach to information seeking amid influenza-like illness outbreaks
Source: Front Public Health. 2025 Mar 24;13:1545942. doi: 10.3389/fpubh.2025.1545942 (PMC11973319; doi:10.3389/fpubh.2025.1545942)
Supplement: Supplementary file 1 [file Table_1.DOCX]

**TABLE**  Chinese stratified sampling size.

|  | The first level | The second level | Population | Percentage^a^ | Sample size | Sample size  (rounded up) |
| --- | --- | --- | --- | --- | --- | --- |
| China | Eastern  Region | Beijing | 21,893,095 | 1.55% | 29.264 | 29 |
|  |  | Tianjin | 13,866,009 | 0.98% | 18.502 | 19 |
|  |  | Hebei | 74,610,235 | 5.29% | 99.686 | 100 |
|  |  | Shanghai | 24,870,895 | 1.76% | 33.228 | 33 |
|  |  | Jiangsu | 84,748,016 | 6.01% | 113.47 | 113 |
|  |  | Zhejiang | 64,567,588 | 4.58% | 86.470 | 86 |
|  |  | Fujian | 41,540,086 | 2.95% | 55.696 | 56 |
|  |  | Shandong | 101,527,453 | 7.20% | 135.936 | 136 |
|  |  | Guangdong | 126,012,510 | 8.94% | 168.787 | 169 |
|  |  | Hainan | 10,081,232 | 0.72% | 13.594 | 14 |
|  | Central Region | Shanxi (山西) | 34,915,616 | 2.48% | 46.822 | 47 |
|  |  | Anhui | 61,027,171 | 4.33% | 81.750 | 82 |
|  |  | Jiangxi | 45,188,635 | 3.21% | 60.605 | 61 |
|  |  | Henan | 99,365,519 | 7.05% | 133.104 | 133 |
|  |  | Hubei | 57,752,557 | 4.10% | 77.408 | 77 |
|  |  | Hunan | 66,444,864 | 4.71% | 88.924 | 89 |
|  | Western Region | Neimenggu | 24,049,155 | 1.71% | 32.284 | 32 |
|  |  | Guangxi | 50,126,804 | 3.56% | 67.213 | 67 |
|  |  | Chongqing | 32,054,159 | 2.27% | 42.857 | 43 |
|  |  | Sichuan | 83,674,866 | 5.94% | 112.147 | 112 |
|  |  | Guizhou | 38,562,148 | 2.74% | 51.731 | 52 |
|  |  | Yunnan | 47,209,277 | 3.35% | 63.248 | 63 |
|  |  | Xizang | 3,648,100 | 0.26% | 4.908 | 5 |
|  |  | Shanxi (陕西) | 39,528,999 | 2.80% | 52.864 | 53 |
|  |  | Gansu | 25,019,831 | 1.77% | 33.417 | 33 |
|  |  | Qinghai | 5,923,957 | 0.42% | 7.929 | 8 |
|  |  | Ningxia | 7,202,654 | 0.51% | 9.628 | 10 |
|  |  | Xinjiang | 25,852,345 | 1.83% | 34.550 | 34 |
|  | Northeast Region | Liaoning | 42,591,407 | 3.02% | 57.017 | 57 |
|  |  | Jilin | 24,073,453 | 1.71% | 32.284 | 32 |
|  |  | Heilongjiang | 31,850,088 | 2.26% | 42.668 | 43 |
| Mainland of China^b^ | | 1,409,778,724 | | 1,888 | | |

^a^ Percentage refers to the proportion of the permanent population of each province, autonomous region, and municipality to the national population.

^b^ The Mainland of China population refers to the population of the 31 provinces, autonomous regions, municipalities in the mainland, excluding active military, Hong Kong, Macao, and Taiwan residents and foreigners living in the 31 provinces, autonomous regions, and municipalities, data are obtained from China’s Seventh National Census (52).
