# Supplementary material for: From fear to facts: a multi-channel approach to information seeking amid influenza-like illness outbreaks
Source: Front Public Health. 2025 Mar 24;13:1545942. doi: 10.3389/fpubh.2025.1545942 (PMC11973319; doi:10.3389/fpubh.2025.1545942)
Supplement: Supplementary file 2 [file Table_2.DOCX]

**TABLE**  Demographic information of respondents (N = 2604)

| **Demographic factors** |  | **n** | **%** |
| --- | --- | --- | --- |
| **Gender** | Male | 1323 | 50.8% |
|  | Female | 1281 | 49.2% |
|  |  |  |  |
| **Age** | 18 - 21 | 193 | 7.4% |
|  | 22 - 25 | 337 | 12.9% |
|  | 26 - 29 | 602 | 23.1% |
|  | 30 - 33 | 672 | 25.8% |
|  | 34 - 37 | 382 | 14.7% |
|  | 38 - 41 | 183 | 7.0% |
|  | 42 - 45 | 114 | 4.4% |
|  | 46 - 49 | 50 | 1.9% |
|  | 50 - 53 | 33 | 1.3% |
|  | 54 - 57 | 27 | 1.0% |
|  | ≥ 58 | 11 | 0.4% |
|  |  |  |  |
| **Education** | No schooling | 0 | 0 |
|  | Primary school | 0 | 0 |
|  | Junior secondary school | 23 | 0.9% |
|  | Senior secondary school | 75 | 2.9% |
|  | Medium vocational education | 35 | 1.3% |
|  | High vocational education | 29 | 1.1% |
|  | College | 342 | 13.1% |
|  | University | 1902 | 73.0% |
|  | Graduate and higher level | 198 | 7.6% |
|  |  |  |  |
| **Occupation** | Unit head | 288 | 11.1% |
|  | Technicians and associates professionals | 1218 | 46.8% |
|  | Clerical support workers | 307 | 11.8% |
|  | Service and sales workers | 307 | 11.8% |
|  | Skilled agriculture, forestry, livestock and fishery workers | 23 | 0.9% |
|  | Craft and related trades workers | 46 | 1.8% |
|  | Others | 398 | 15.3% |
|  | Retiree | 9 | 0.3% |
|  | Unemployed | 8 | 0.3% |
|  |  |  |  |
| **Region** | Beijing | 56 | 2.2% |
|  | Tianjin | 26 | 1.0% |
|  | Hebei | 119 | 4.6% |
|  | Shanghai | 51 | 2.0% |
|  | Jiangsu | 145 | 5.6% |
|  | Zhejiang | 109 | 4.2% |
|  | Fujian | 86 | 3.3% |
|  | Shandong | 176 | 6.8% |
|  | Guangdong | 227 | 8.7% |
|  | Hainan | 17 | 0.7% |
|  | Shanxi | 56 | 2.2% |
|  | Anhui | 123 | 4.7% |
|  | Jiangxi | 90 | 3.5% |
|  | Henan | 185 | 7.1% |
|  | Hubei | 113 | 4.3% |
|  | Hunan | 120 | 4.6% |
|  | Heilongjiang | 63 | 2.4% |
|  | Jilin | 47 | 1.8% |
|  | Liaoning | 79 | 3.0% |
|  | Neimenggu | 42 | 1.6% |
|  | Guangxi | 86 | 3.3% |
|  | Chongqing | 59 | 2.3% |
|  | Sichuan | 159 | 6.1% |
|  | Guizhou | 79 | 3.0% |
|  | Yunnan | 85 | 3.3% |
|  | Xizang | 11 | 0.4% |
|  | Shanxi | 72 | 2.8% |
|  | Gansu | 51 | 2.0% |
|  | Qinghai | 15 | 0.6% |
|  | Ningxia | 13 | 0.5% |
|  | Xinjiang | 44 | 1.7% |
|  |  |  |  |
| **Salary (RMB/ month)** | 0 | 52 | 2.0% |
|  | 1 - 2500 | 172 | 6.6% |
|  | 2501 - 5000 | 501 | 19.2% |
|  | 5001 - 7500 | 474 | 18.2% |
|  | 7501 - 10000 | 756 | 29.0% |
|  | 10001 - 12500 | 252 | 9.7% |
|  | 12501 - 15000 | 181 | 7.0% |
|  | 15001 - 17500 | 41 | 1.6% |
|  | 17501 - 20000 | 90 | 3.5% |
|  | 20001 - 22500 | 9 | 0.3% |
|  | 22501 - 25000 | 28 | 1.1% |
|  | ≥ 25001 | 48 | 1.8% |
