# Supplementary material for: From fear to facts: a multi-channel approach to information seeking amid influenza-like illness outbreaks
Source: Front Public Health. 2025 Mar 24;13:1545942. doi: 10.3389/fpubh.2025.1545942 (PMC11973319; doi:10.3389/fpubh.2025.1545942)
Supplement: Supplementary file 3 [file Table_3.DOCX]

**TABLE** Measured constructs and specific items

| **Constructs** | **Sources** | **Number of measurement items** | **Level of**  **measurement** |
| --- | --- | --- | --- |
| **Risk perception** | Yang et al., 2014 (40) | 8 | Interval |
| **Affective responses** | Jin & Lane, 2022 (12) | 7 | Interval |
| **Information subjective norms (ISN)** | Yang et al., 2014 (40) | 5 | Interval |
| **Information insufficiency** | Yang & Zhuang, 2020 (13) | 2 | Interval |
| **Channel complementarity beliefs** | Rains & Ruppel, 2016 (55) | 32 | Interval |
| **Intent for multichannel risk information seeking** | Hwang & Jeong, 2020 (9);  Yang et al., 2011 (15);  Jia et al., 2021(29). | 4 | Ratio |
