# Supplementary material for: From fear to facts: a multi-channel approach to information seeking amid influenza-like illness outbreaks
Source: Front Public Health. 2025 Mar 24;13:1545942. doi: 10.3389/fpubh.2025.1545942 (PMC11973319; doi:10.3389/fpubh.2025.1545942)
Supplement: Supplementary file 4 [file Table_4.DOCX]

**TABLE** Descriptive statistics and confirmatory factor analysis (CFA) of measured variables.

| **Item name** | | **Answer range** | | **Mean** | | **Median** | **SD** | | **Factor**  **loading** |
| --- | --- | --- | --- | --- | --- | --- | --- | --- | --- |
| **Risk perception (α = 0.90)** | 1-6 | | 4.89 | | 5.00 | | 0.85 |  | |
| RP1 |  | |  | |  | |  | 0.73 | |
| RP2 |  | |  | |  | |  | 0.77 | |
| RP3 |  | |  | |  | |  | 0.70 | |
| RP4 |  | |  | |  | |  | 0.57 | |
| RP6 |  | |  | |  | |  | 0.81 | |
| RP7 |  | |  | |  | |  | 0.84 | |
| RP8 |  | |  | |  | |  | 0.71 | |
| RP9 |  | |  | |  | |  | 0.56 | |
| **Negative affective response (α = 0.81)** | 1-6 | | 4.29 | | 4.50 | | 0.92 |  | |
| NAR1 |  | |  | |  | |  | 0.80 | |
| NAR2 |  | |  | |  | |  | 0.66 | |
| NAR3 |  | |  | |  | |  | 0.68 | |
| NAR4 |  | |  | |  | |  | 0.73 | |
| **Positive affective response (α = 0.82)** | 1-6 | | 1.95 | | 1.67 | | 0.99 |  | |
| PAR6 |  | |  | |  | |  | 0.81 | |
| PAR7 |  | |  | |  | |  | 0.86 | |
| PAR9 |  | |  | |  | |  | 0.67 | |
| **Information insufficiency (r = .75 (p < 0.01))** | 1-6 | | 5.10 | | 5.00 | | 0.71 |  | |
| II1 |  | |  | |  | |  | 0.72 | |
| II2 |  | |  | |  | |  | 0.76 | |
| **Information subjective norms (α = 0.84)** | 1-6 | | 4.80 | | 5.00 | | 0.72 |  | |
| ISN1 |  | |  | |  | |  | 0.71 | |
| ISN2 |  | |  | |  | |  | 0.78 | |
| ISN3 |  | |  | |  | |  | 0.70 | |
| ISN4 |  | |  | |  | |  | 0.69 | |
| ISN5 |  | |  | |  | |  | 0.69 | |
| **Channel complementarity beliefs (α = 0.92)** | 1-6 | | 4.50 | | 4.53 | | 0.52 |  | |
| Access to medical expertise belief of interpersonal source |  | |  | |  | |  |  | |
| INME1 |  | |  | |  | |  | 0.71 | |
| INME2 |  | |  | |  | |  | 0.79 | |
| Tailorability belief of interpersonal source |  | |  | |  | |  |  | |
| INTA1 |  | |  | |  | |  | 0.78 | |
| INTA2 |  | |  | |  | |  | 0.78 | |
| Convenience belief of interpersonal source |  | |  | |  | |  |  | |
| INCO1 |  | |  | |  | |  | 0.78 | |
| INCO2 |  | |  | |  | |  | 0.78 | |
| Anonymity belief of interpersonal source |  | |  | |  | |  |  | |
| INAN1 |  | |  | |  | |  | 0.68 | |
| INAN2 |  | |  | |  | |  | 0.80 | |
| Access to medical expertise belief of traditional source |  | |  | |  | |  |  | |
| TAME1 |  | |  | |  | |  | 0.65 | |
| TAME2 |  | |  | |  | |  | 0.81 | |
| Tailorability belief of traditional source |  | |  | |  | |  |  | |
| TRTA1 |  | |  | |  | |  | 0.79 | |
| TRTA2 |  | |  | |  | |  | 0.80 | |
| Convenience belief of traditional source |  | |  | |  | |  |  | |
| TRCO1 |  | |  | |  | |  | 0.81 | |
| TRCO2 |  | |  | |  | |  | 0.80 | |
| Anonymity belief of traditional source |  | |  | |  | |  |  | |
| TRAN1 |  | |  | |  | |  | 0.74 | |
| TRAN2 |  | |  | |  | |  | 0.76 | |
| Access to medical expertise belief of search engines |  | |  | |  | |  |  | |
| SEME1 |  | |  | |  | |  | 0.70 | |
| SEME2 |  | |  | |  | |  | 0.76 | |
| Tailorability belief of search engines |  | |  | |  | |  |  | |
| SETA1 |  | |  | |  | |  | 0.76 | |
| SETA2 |  | |  | |  | |  | 0.76 | |
| Anonymity belief of search engines |  | |  | |  | |  |  | |
| SEAN1 |  | |  | |  | |  | 0.79 | |
| SEAN2 |  | |  | |  | |  | 0.79 | |
| Convenience belief of search engines |  | |  | |  | |  |  | |
| SECO1 |  | |  | |  | |  | 0.73 | |
| SECO2 |  | |  | |  | |  | 0.73 | |
| Access to medical expertise belief of social media |  | |  | |  | |  |  | |
| SOME1 |  | |  | |  | |  | 0.72 | |
| SOME2 |  | |  | |  | |  | 0.77 | |
| Tailorability belief of social media |  | |  | |  | |  |  | |
| SOTA1 |  | |  | |  | |  | 0.77 | |
| SOTA2 |  | |  | |  | |  | 0.76 | |
| Anonymity belief of social media |  | |  | |  | |  |  | |
| SOAN1 |  | |  | |  | |  | 0.82 | |
| SOAN2 |  | |  | |  | |  | 0.76 | |
| Convenience belief of social media |  | |  | |  | |  |  | |
| SOCO1 |  | |  | |  | |  | 0.77 | |
| SOCO2 |  | |  | |  | |  | 0.75 | |
| **Intent for multi-channel information seeking** | 0-10 | | 7.13 | | 7.25 | | 1.34 |  | |
